# *PPARG, TMEM163, UBE2E2,* and *WFS1* Gene Polymorphisms Are Not Significant Risk Factors for Gestational Diabetes in the Polish Population

**DOI:** 10.3390/jpm12020243

**Published:** 2022-02-08

**Authors:** Przemysław Ustianowski, Damian Malinowski, Krzysztof Safranow, Violetta Dziedziejko, Maciej Tarnowski, Andrzej Pawlik

**Affiliations:** 1 Department of Obstetrics and Gynecology, Pomeranian Medical University, 70-111 Szczecin, Poland; przemyslaw.ustianowski@pum.edu.pl; 2 Department of Experimental and Clinical Pharmacology, Pomeranian Medical University, 70-111 Szczecin, Poland; damian.malinowski@pum.edu.pl; 3 Department of Biochemistry and Medical Chemistry, Pomeranian Medical University, 70-111 Szczecin, Poland; viola@pum.edu.pl (V.D.); chrissaf@mp.pl (K.S.); 4 Department of Physiology, Pomeranian Medical University, 70-111 Szczecin, Poland; maciejt@pum.edu.pl

**Keywords:** gestational diabetes, polymorphism, genetics

## Abstract

Gestational diabetes mellitus (GDM) is a common disorder that occurs in pregnant women, leading to many maternal and neonatal complications. The pathogenesis of GDM is complex and includes risk factors, such as: age, obesity, and family history of diabetes. Studies have shown that genetic factors also play a role in the pathogenesis of GDM. The present study investigated whether polymorphisms in the *PPARG* (rs1801282), *TMEM163* (rs6723108 and rs998451), *UBE2E2* (rs6780569), and *WFS1* (rs4689388) genes are risk factors for the development of GDM and whether they affect selected clinical parameters in women with GDM. This study included 204 pregnant women with GDM and 207 pregnant women with normal glucose tolerance (NGT). The diagnosis of GDM was based on a 75 g oral glucose tolerance test (OGTT) at 24–28 weeks gestation, according to the International Association of Diabetes and Pregnancy Study Groups (IADPSG) criteria. There were no statistically significant differences in the distribution of polymorphisms studied between women with GDM and pregnant women with normal carbohydrate tolerance, which suggests that these polymorphisms are not risk factors for GDM. We also examined the associations between studied gene polymorphisms and clinical parameters: fasting glucose, daily insulin requirement, body mass before pregnancy, body mass at birth, body mass increase during pregnancy, BMI before pregnancy, BMI at birth, BMI increase during pregnancy, new-born body mass, and APGAR score in women with GDM. We observed lower BMI values before pregnancy and at birth in women with *PPARG* rs17036160 TT genotype. The results of this study suggest that the *PPARG* (rs1801282), *TMEM163* (rs6723108 and rs998451), *UBE2E2* (rs6780569), and *WFS1* (rs4689388) gene polymorphisms are not significant risk factors for GDM development in the Polish population and do not affect the clinical parameters in women with GDM; only rs1801282 of the *PPARG* gene may influence BMI values in women with GDM.

## 1. Introduction

Gestational diabetes mellitus (GDM) is carbohydrate intolerance occurring in pregnant women. GDM may lead to various metabolic complications; therefore, factors causing a predisposition for the development of GDM are being investigated [1]. GDM occurs in about 14% of pregnancies worldwide, which represents about 18 million cases per year [2]. GDM is characterized by the inability of pancreatic beta cells to respond adequately to increased insulin requirements during pregnancy, resulting in varying degrees of hyperglycemia [2]. Pancreatic beta cell dysfunction is considered to be the result of prolonged, excessive insulin production. The pathogenesis of GDM is complex and includes risk factors, such as age, obesity, and family history of diabetes [2,3,4,5]. In women with GDM, both impaired insulin secretion and insulin resistance was observed [2,3,4,6]. Each of the risk factors are associated with impaired insulin production or reduced insulin sensitivity. For example, overweight and obesity are associated with excessive insulin production and chronic inflammation.

GDM is characterized by chronic inflammation that negatively influences the fetus. Elevated expression of inflammatory mediators was also found in the placentas of women with GDM, especially those with obesity [5]. Hyperglycemia is associated with a well-documented array of adverse maternal and fetal consequences. Children born to mothers with GDM are at increased risk for a number of direct complications including preterm birth, macrosomia, respiratory failure, joint abnormalities, and neonatal hypoglycemia [2,3,4]. Women with GDM have an increased risk of a number of serious perinatal complications, including gestational hypertension, pre-eclampsia, preterm birth, and the development of type 2 diabetes (T2DM). Studies suggest that the risk of developing T2DM in women with GDM may be up to seven times higher than in women with normal glucose tolerance. Approximately 60% of women with a history of GDM develop T2DM. The observed insulin resistance and impaired insulin secretion are similar to those in T2DM [3,5]. The significant prevalence of T2DM in women with previous GDM raises the possibility that there is a common genetic basis. Many genes related to pancreatic beta cell development, function, and survival have been identified as affecting T2DM and GDM risk in association studies. 

Previous studies have shown that some genetic loci causing a predisposition for the development of type 2 diabetes mellitus may also cause a predisposition for GDM [7,8]. 

The peroxisome proliferator-activated receptors-γ (PPARG) is the transcription factor belonging to the nuclear hormone receptor superfamily. PPARG regulates carbohydrate and lipid metabolism, fatty acid transport, adipocyte differentiation, and inflammation [9]. The expression of PPARG was detected mainly in adipose tissue. Previous studies indicated a significant role of PPARG and *PPARG* gene polymorphisms in the pathogenesis of type 2 diabetes [10,11,12].

TMEM163 is a 31.5 kDa protein that binds cations, such as zinc. Previous studies suggest the involvement of TMEM163 in insulin secretion and type 2 diabetes pathogenesis. It has been shown that TMEM163 expression in MIN6 cells correlated with decreased insulin secretion and expression of genes involved in glucose metabolism [13]. Moreover, TMEM163 mRNA expression in human pancreatic tissue from patients with type 2 diabetes was significantly increased [13,14]. TMEM163 was associated with a high glycemic index and fasting plasma insulin level.

UBE2E2 encodes ubiquitin-conjugating enzyme E2E2, which plays an important role in the synthesis and secretion of insulin. UBE2E2 is expressed in the pancreas, liver, and adipose tissue. Previous studies indicated the role of UBE2E2 and *UBE2E2* gene polymorphisms in the pathogenesis of type 2 diabetes [15,16]. 

The *WFS1* gene is associated with Wolfram syndrome (diabetes insipidus, diabetes mellitus, optic atrophy, and deafness). Recently, this gene was investigated as a genetic factor for the predisposition to type 1 and type 2 diabetes development. *WFS1* was included as a candidate gene evaluated for association with type 2 diabetes [17,18].

In this study, we examined polymorphisms in the *PPARG* (rs1801282), *TMEM163* (rs6723108 and rs998451), *UBE2E2* (rs6780569), and *WFS1* (rs4689388) genes in women with GDM. We aimed to investigate whether these polymorphisms affect the risk of developing GDM and whether they influence clinical parameters in women with GDM. We compared the distribution of polymorphisms studied between women with GDM and women with normal glucose tolerance during pregnancy, and assessed the correlations between the genotypes studied and selected clinical parameters.

## 2. Materials and Methods

### 2.1. Participants

This case–control association study included 204 pregnant women with GDM and 207 pregnant women with normal glucose tolerance (NGT) treated in the Department of Obstetrics and Gynecology, Pomeranian Medical University, Szczecin, Poland. Women with multi-fetal pregnancy, other complications of pregnancy, and those who did not give their consent for the study were not included. The diagnosis of GDM was based on a 75 g oral glucose tolerance test (OGTT) at 24–28 weeks gestation, according to the International Association of Diabetes and Pregnancy Study Groups (IADPSG) criteria [19]. The diagnosis of GDM was made when one of the following plasma glucose values in the OGTT was met or exceeded: fasting plasma glucose of 92 mg/dL (5.1 mmol/L), 1 h plasma glucose of 180 mg/dL (10.0 mmol/L), or 2 h plasma glucose of 153 mg/dL (8.5 mmol/L). Exclusion criteria were: type 1 and type 2 diabetes, autoimmune and inflammatory diseases, neoplasmatic diseases, and chronic infections. All pregnancies were achieved by natural conception. Among the pregnant women with GDM, 78% of them were treated with diet control alone throughout their pregnancies, while the remaining 22% were treated with diet control and insulin until delivery. All pregnant women were without any acute or chronic complications, such as diabetic ketoacidosis or other disorders affecting glucose metabolism. The subjects were educated about this study. Written informed consent was obtained from all subjects. The study was approved by the Ethics Committee of Pomeranian Medical University, Szczecin, Poland (KB-0012/40/14). 

### 2.2. Methods

All samples were genotyped in duplicate using allelic discrimination assays with TaqMan^®^ probes (Applied Biosystems, Carlsbad, CA, USA) on a 7500 Fast Real-Time PCR Detection System (Applied Biosystems). In order to discriminate the polymorphisms, we employed TaqMan^®^ Pre-Designed SNP Genotyping Assays, including appropriate primers and fluorescently labelled (FAM and VIC) MGB™ probes to detect the alleles. 

### 2.3. Statistical Analysis

The consistency of the genotype distribution with Hardy–Weinberg equilibrium (HWE) was assessed using the exact test. A chi-square test was used to compare the genotype and allele distributions between the groups. Distributions of most of the quantitative variables were significantly different from normal distribution (Shapiro–Wilk test), so they were compared between the genotype groups using the non-parametric Mann–Whitney U test. Data were presented as median and interquartile range (IQR). Multivariate logistic regression model adjusted for age and pre-gestational BMI, which are known risk factors of GDM, was used to find whether each polymorphic allele is an independent risk factor of GDM. *p*-values < 0.05 were considered statistically significant. The study with 204 patients and 207 controls has the statistical power sufficient to detect with 80% probability true effect sizes corresponding to odds ratio (OR) for allelic association equal to 0.20 or 2.48 for *UBE2E2* rs6780569, 0.35 or 2.09 for *PPARG* rs17036160, 0.52 or 1.78 for *TMEM163* rs6723108 and rs998451, and 0.57 or 1.74 for *WFS1* rs4689388.

## 3. Results

The distributions of the studied polymorphisms were in the HWE (*p* > 0.05). The distributions of studied polymorphisms in women with GDM and control women are shown in Table 1. As shown in Table 1, there are no statistically significant differences in the distribution of studied gene polymorphisms between women with GDM and pregnant women with normal carbohydrate tolerance. None of the five analyzed SNPs were a significant independent predictor of GDM in logistic regression models adjusted for age and BMI (*p* > 0.05), while older age and higher BMI were, as expected, strongly associated with GDM risk (*p* < 0.00003 for age and *p* < 0.0002 for BMI).

We also examined the associations between the studied gene polymorphisms and clinical parameters, such as fasting glucose, daily insulin requirement, body mass before pregnancy, body mass at birth, body mass increase during pregnancy, BMI before pregnancy, BMI at birth, BMI increase during pregnancy, new-born body mass, and APGAR score in women with GDM (Table 2, Table 3, Table 4, Table 5 and Table 6).

The majority of associations between the above parameters and studied polymorphisms were statistically non-significant. We only observed lower BMI values before pregnancy and at birth in women with *PPARG* rs17036160 TT genotype. 

## 4. Discussion

GDM is a disorder of carbohydrate metabolism that occurs in pregnant women. The pathogenesis of this disease is complex. GDM is caused by impaired insulin secretion in pancreatic beta islands, as well as chronic inflammation and tissue insulin resistance. Several environmental and genetic factors cause a predisposition to GDM, including a family history of type 2 diabetes, obesity, female age, and pre-pregnancy carbohydrate metabolism disorders [1,3]. Due to the numerous maternal and fetal complications of GDM, factors that increase the risk of developing this disease are being investigated. Identification of these factors could be helpful in early diagnosis and in identifying women who are at increased risk of GDM developing. This would allow for earlier implementation of prevention and treatment. A number of genetic factors are currently being considered that may cause a predisposition to GDM [2,3]. Since the pathogenesis of GDM is similar to that of type 2 diabetes, genes that increase the risk of type 2 diabetes have been studied [5,8]. These factors may include polymorphisms of genes affecting pancreatic beta-cell function, insulin resistance, carbohydrate and lipid metabolism, and inflammation [5,7]. These polymorphisms can affect gene expression and, thus, the amount of protein synthesis they regulate. Inter-individual differences in the expression of these genes may affect processes involved in insulin release and carbohydrate metabolism and, thereby, increase the risk of GDM. 

In this study, we examined the association between *PPARG* (rs1801282), *TMEM163* (rs6723108, rs998451), *UBE2E2* (rs6780569), and *WFS1* (rs4689388) gene polymorphisms and GDM. To date, the association between these polymorphisms and GDM in the Caucasian population has not been investigated. Our results did not show that these gene polymorphisms were associated with the risk of developing GDM. We also examined the association between the polymorphisms studied and clinical parameters in women. These associations were not statistically significant. We only observed a statistically significant association between *PPARG* gene polymorphism and BMI values before pregnancy and at birth. 

Peroxisome proliferator-activated receptor γ (PPARγ) is a nuclear hormone receptor expressed mainly in adipose tissue [20]. Its activation causes binding of specific DNA elements and induction of a transcriptional cascade that leads to adipocyte differentiation and increased insulin sensitivity. Previous studies indicate an important role for PPARG in diseases, such as obesity and diabetes [20,21,22,23,24]. In obesity, PPARG regulates adipocyte maturation and differentiation. It is an essential factor for the adipocyte differentiation process and, thus, acts as a regulator of adipogenesis. In addition, PPARG plays an important role in the process of insulin resistance. Mice lacking the PPARG gene in muscle are insulin resistant. In adipose tissue, PPARG deletion leads to lipoatrophy and insulin resistance (IR) [20]. Lendvai et al. demonstrated that maternal nutrition can affect PPARG gene methylation and foetal and placenta development [25]. Wojcik et al. have shown the correlation between leukocyte PPARG overexpression and hyperglycemia, suggesting that PPARG mRNA expression in these cells might be up-regulated in high-glucose conditions in GDM patients during gestation [26]. 

Previous studies investigated the associations between *PPARG* gene rs1801282 polymorphism and GDM in various populations. In the meta-analysis, Wu et al. have shown that the rs1801282 polymorphism in the *PPARG* gene correlated significantly with a risk of GDM in Asian populations [27]. Additionally, the study conducted in a Brazilian population suggests a significant association between *PPARG* gene rs1801282 polymorphism and GDM [28]. In the meta-analysis by Wang et al., *PPARG* gene rs1801282 polymorphism was associated significantly with the GDM risks in East Asians, while no significant associations were detected among Caucasian and Middle Eastern populations [29]. The meta-analysis by Mao et al., including 11 studies, showed a lack of statistically significant association between *PPARG* gene rs1801282 polymorphism and GDM both in Caucasian and East Asian populations [30].

TMEM163 is a cation transport protein involved in insulin secretion in pancreatic beta-cells. It has been shown that patients with T2DM may have a mutation in this gene leading to reduced insulin secretion [13]. *TMEM163* gene polymorphisms were investigated in T2DM patients in various populations, however the results are inconsistent. Tabassum et al. suggest an association between *TMEM163* gene rs998451 and rs6723108 polymorphisms and type 2diabetes and insulin secretion in Indian population [31]. However, these associations were not confirmed in another study conducted in a northwestern India population [32]. Bai et al. indicated that the *TMEM163* gene rs6723108 polymorphism is associated with T2DM in Mongolian but not Caucasian populations [33]. Tabassum et al. have shown that *TMEM163* gene variants showed association with decreased fasting plasma insulin and insulin resistance, indicating an effect through impaired insulin secretion [31]. Tan et al. examined the association between *TMEM163* gene rs998451 polymorphisms and the risk of GDM, as well as fasting insulin levels [34]. The results suggest a lack of a statistically significant association between *TMEM163* gene rs998451 polymorphism and GDM in Chinese populations [34]. The results of our study also suggest a lack of statistically significant association between *TMEM163* gene rs998451 and rs6723108 polymorphisms and risk of GDM, as well as some clinical parameters in women with GDM in the Polish population. 

The ubiquitin-conjugating enzyme E2E2 (*UBE2E2* gene) plays an important role in insulin synthesis and secretion. Previous studies indicated the association between *UBE2E2* gene polymorphism and diabetes in various population [15]. Kazakova et al. suggests the association between *UBE2E2* gene rs7612463 polymorphism and T2DM in the Chinese population [15]. Similar results obtained Zeng et al., who suggest the association between *UBE2E2* gene polymorphism, obesity, and T2DM [35]. Moreover, *UBE2E2* gene rs7119 polymorphisms correlated with insulin release after glucose stimulation in elderly Chinese Han individuals [16]. These observations were not confirmed in other Asian populations (Japanese, Thai, and Saudi) where no statistically significant associations between *UBE2E2* gene polymorphisms and T2DM were found [36,37,38]. 

Kim et al. examined *UBE2E2* gene rs6780569 and rs7612463 polymorphisms in Korean women with GDM [39]. These authors suggest that these polymorphisms are associated with fasting plasma glucose. Moreover, rs7612463 polymorphisms were associated with GDM risk in Korean women [39]. The results of our study suggest lack of statistically significant association between *UBE2E2* gene rs6780569 polymorphism and fasting plasma glucose and GDM risk in our Caucasian population.

*WFS1* gene is considered as genetic factor causing a predisposition to type 1 and type 2 diabetes in different populations [40,41]. The protein encoded by the *WFS1* gene—Wolframin—causes increased apoptosis and dysfunction of pancreatic beta cells. A number of studies have investigated the association between *WFS1* gene polymorphism and insulin secretion, insulinemia, insulin sensitivity, as well as risk of hyperglycemia and T2DM [42,43,44,45]. The results of studies suggest that the *WFS1* gene may be the candidate gene for type 2 diabetes [41,46]. Long et al. suggest that *WFS1* gene polymorphisms may be associated with T2DM risk in African-American populations [47]. To date, no association of the *WFS1* gene polymorphism with GDM has been demonstrated [48]. The results of our study suggest lack of statistically significant association between *WFS1* gene polymorphism and GDM in the Polish population.

The results of our study suggest a lack of statistically significant associations between the studied gene polymorphisms and GDM. We have only shown the association between the *PPARG* gene rs1801282 polymorphism and BMI values in women with GDM. Studies suggest that some polymorphisms of genes associated with the risk of type 2 diabetes have an impact on the occurrence of GDM. However, this depends on the population studied and the possible association between these polymorphisms and other genes affecting carbohydrate metabolism and predisposition to GDM. Additionally, important is the influence of many environmental factors such as obesity, as well as the diet of pregnant women. The study results suggest that the influence of genetic polymorphisms on the risk of GDM is small. Therefore, the effect of gene polymorphisms on the risk of GDM should be considered together with other environmental factors that increase the risk of developing this disease. It is not excluded that the polymorphisms studied have a very small effect on the risk of GDM, but it would be detectable if a very large number of cases were studied. The lack of an effect of the polymorphisms studied on GDM risk and clinical parameters also does not exclude the role of the genes investigated in the pathogenesis of GDM. Understanding the role of *PPARG*, *TMEM163*, *UBE2E2*, and *WFS1* genes in the pathogenesis of GDM requires further studies.

## 5. Conclusions

The results of this study suggest that *PPARG* (rs1801282), *TMEM163* (rs6723108 and rs998451), *UBE2E2* (rs6780569), and *WFS1* (rs4689388) gene polymorphisms are not significant risk factors for GDM development in the Polish population and do not affect the clinical parameters in women with GDM, only rs1801282 of *PPARG* gene may influence BMI values in women with GDM.

## Figures and Tables

**Table 1:** Distribution of *PPARG, TMEM163, UBE2E2, and WFS1* genotypes and alleles in women with GDM and control group.

	Control Group		GDM		*p* Value^^^		OR (95% CI)	*p* Value ^^^
	*n*	%	*n*	%				
***PPARG* rs17036160 genotype**								
CC	159	76.81%	156	76.47%	0.94	TT + CT vs. CC	1.02 (0.65–1.61)	0.93
CT	43	20.77%	44	21.57%		TT vs. CT + CC	0.81 (0.21–3.05)	0.75
TT	5	2.42%	4	1.96%		TT vs. CC	0.82 (0.21–3.09)	0.76
						CT vs. CC	1.04 (0.65–1.68)	0.86
						TT vs. CT	0.78 (0.20–3.11)	0.73
**Allele**								
C	361	87.20%	356	87.25%		T vs. C	0.99 (0.66–1.50)	0.98
T	53	12.80%	52	12.75%				
								
***TMEM163* rs6723108 genotype**								
TT	92	44.44%	98	48.04%	0.76	GG + GT vs. TT	0.87 (0.59–1.28)	0.46
GT	93	44.93%	86	42.16%		GG vs. GT + TT	0.91 (0.48–1.73)	0.78
GG	22	10.63%	20	9.80%		GG vs. TT	0.85 (0.44–1.67)	0.64
						GT vs. TT	0.87 (0.58–1.31)	0.50
						GG vs. GT	0.98 (0.50–1.93)	0.96
**Allele**								
T	277	66.91%	282	69.12%		G vs. T	0.90 (0.67–1.21)	0.50
G	137	33.09%	126	30.88%				
								
***TMEM163* rs998451 genotype**								
GG	94	45.41%	103	50.49%	0.59	AA + GA vs. GG	0.82 (0.55–1.20)	0.30
GA	91	43.96%	81	39.71%		AA vs. GA + GG	0.91 (0.48–1.73)	0.78
AA	22	10.63%	20	9.80%		AA vs. GG	0.83 (0.43–1.62)	0.58
						GA vs. GG	0.81 (0.54–1.22)	0.32
						AA vs. GA	1.02 (0.52–2.01)	0.95
**Allele**								
G	279	67.39%	287	70.34%		A vs. G	0.87 (0.65–1.17)	0.36
A	135	32.61%	121	29.66%				
								
***UBE2E2* rs6780569 genotype**								
GG	180	86.96%	176	86.27%	0.89	AA + GA vs. GG	1.06 (0.60–1.87)	0.84
GA	25	12.08%	25	12.25%		AA vs. GA + GG	1.53 (0.25–9.25)	0.64
AA	2	0.97%	3	1.47%		AA vs. GG	1.53 (0.25–9.29)	0.64
						GA vs. GG	1.02 (0.57–1.85)	0.94
						AA vs. GA	1.50 (0.23–9.76)	0.67
**Allele**								
G	385	93.00%	377	92.40%		A vs. G	1.09 (0.65–1.85)	0.74
A	29	7.00%	31	7.60%				
								
***WFS1* rs4689388** **genotype**								
AA	52	25.12%	64	31.37%	0.24	GG + GA vs. AA	0.73 (0.48–1.13)	0.16
GA	114	55.07%	96	47.06%		GG vs. GA + AA	1.11 (0.69–1.80)	0.66
GG	41	19.81%	44	21.57%		GG vs. AA	0.87 (0.50–1.53)	0.63
						GA vs. AA	0.68 (0.69–1.20)	0.10
						GG vs. GA	1.27 (0.43–1.08)	0.35
**Allele**								
A	218	52.66%	224	54.90%		G vs. A	0.91 (0.77–2.11)	0.52
G	196	47.34%	184	45.10%				

^ χ^2^ test. HWE: control group *p* = 0.34, GDM group *p* = 0.75 for *PPARG* rs17036160. HWE: control group *p*= 0.88, GDM group *p* = 0.87 for *TMEM163* rs6723108. HWE: control group *p* = 1.00, GDM group *p* = 0.50 for *TMEM163* rs998451. HWE: control group *p* = 0.26, GDM group *p* = 0.09 for *UBE2E2* rs6780569. HWE: control group *p*= 0.16, GDM group *p* = 0.48 for *WFS1* rs4689388.

**Table 2:** Clinical parameters of women with GDM stratified according to *PPARG* rs17036160 genotype.

Parameters	*PPARG* rs17036160 Genotype					
	CC *n* = 154	CT *n* = 44	TT *n* = 4	CC vs. CT	CC vs. TT	CT vs. TT
	Median (IQR)	Median (IQR)	Median (IQR)	*p* ^&^		
Fasting glucose [mg/dl]	98.3 (93.0–105.0)	99.0 (94.0–105.0)	97.5 (91.5–98.5)	0.97	0.34	0.32
Daily insulin requirement [unit]	0.0 (0.0–6.5)	0.0 (0.0–0.0)	0.0 (0.0–0.0)	0.10	0.21	0.39
Body mass before pregnancy [kg]	65.0 (56.5–76.0)	65.0 (58.5–73.5)	55.5 (53.0–58.5)	0.85	0.05	0.044
Body mass at birth [kg]	77.0 (67.0–90.0)	76.0 (68.0–84.0)	65.5 (61.5–67.0)	0.93	0.024	0.012
Body mass increase during pregnancy [kg]	11.0 (7.0–14.0)	11.0 (8.0–13.5)	7.0 (7.0–10.0)	0.93	0.30	0.20
BMI before pregnancy [kg/m^2^]	24.2 (21.0–28.5)	22.8 (21.4–26.3)	20.3 (18.8–21.6)	0.26	0.021	0.032
BMI at birth [kg/m^2^]	28.4 (25.2–33.0)	26.7 (25.-30.1)	23.9 (22.0–24.6)	0.21	0.010	0.011
BMI increase during pregnancy [kg/m^2^]	3.8 (2.7–5.2)	3.6 (2.8–5.0)	2.6 (2.5–3.7)	0.76	0.25	0.16
Newborn body mass [g]	3365 (2985–3685)	3205 (2750–3553)	3305 (3205–3425)	0.17	0.88	0.63
APGAR [0–10]	10.0 (10.0–10.0)	10.0 (10.0–10.0)	10.0 (10.0–10.0)	0.95	0.46	0.48

BMI—body mass index. IQR—Interquartile Range. **^&^** Mann–Whitney U test.

**Table 3:** Clinical parameters of women with GDM stratified according to *TMEM163* rs6723108 genotype.

Parameters	*TMEM163* rs6723108 Genotype					
	TT *n* = 98	GT *n* = 86	GG *n* = 20	TT vs. GT	TT vs. GG	GT vs. GG
	Median (IQR)	Median (IQR)	Median (IQR)	*p* ^&^		
Fasting glucose [mg/dl]	98.0 (94.0–105.0)	98.5 (92.0–105.0)	102.5 (96.0–106.5)	0.86	0.27	0.25
Daily insulin requirement [unit]	0.0 (0.0–0.0)	0.0 (0.0–0.0)	0.0 (0.0–15.0)	0.92	0.079	0.093
Body mass before pregnancy [kg]	65.0 (56.0–76.0)	64.0 (57.0–73.0)	68.5 (59.0–90.0)	0.85	0.17	0.17
Body mass at birth [kg]	76.0 (67.0–90.0)	75.0 (68.0–87.0)	80.0 (70.0–97.5)	0.85	0.15	0.17
Body mass increase during pregnancy [kg]	11.0 (7.0–14.0)	11.0 (8.0–14.0)	9.5 (7.0–14.0)	0.93	0.47	0.48
BMI before pregnancy [kg/m^2^]	23.7 (20.7–28.3)	23.1 (21.1–26.4)	24.6 (21.4–31.0)	0.61	0.29	0.22
BMI at birth [kg/m^2^]	28.1 (25.0–32.6)	27.6 (25.0–31.1)	28.2 (25.4–35.6)	0.58	0.32	0.22
BMI increase during pregnancy [kg/m^2^]	3.9 (2.7–5.3)	3.7 (2.7–5.1)	3.4 (2.5–4.9)	0.71	0.38	0.42
Newborn body mass [g]	3280 (2900–3600)	3345 (2970–3700)	3408 (3165–3683)	0.58	0.32	0.62
APGAR [0–10]	10.0 (10.0–10.0)	10.0 (10.0–10.0)	10.0 (10.0–10.0)	0.87	0.086	0.093

BMI—body mass index. IQR—Interquartile Range. **^&^** Mann–Whitney U test.

**Table 4:** Clinical parameters of women with GDM stratified according to *TMEM163* rs998451 genotype.

Parameters	*TMEM163* rs998451 Genotype					
	GG *n* = 103	GA *n* = 81	AA *n* = 20	GG vs. GA	GG vs. AA	GA vs. AA
	Median (IQR)	Median (IQR)	Median (IQR)	*p* ^&^		
Fasting glucose [mg/dl]	98.5 (94.0–106.0)	98.0 (92.0–104.0)	102.5 (96.0–106.5)	0.39	0.37	0.17
Daily insulin requirement [unit]	0.0 (0.0–4.0)	0.0 (0.0–0.0)	0.0 (0.0–15.0)	0.60	0.11	0.060
Body mass before pregnancy [kg]	65.0 (56.0–76.0)	64.0 (57.0–72.0)	68.5 (59.0–90.0)	0.91	0.17	0.17
Body mass at birth [kg]	76.0 (67.0–90.0)	75.0 (67.0–85.0)	80.0 (70.0–97.5)	0.99	0.16	0.16
Body mass increase during pregnancy [kg]	11.0 (7.0–14.0)	11.0 (8.0–13.0)	9.5 (7.0–14.0)	0.97	0.48	0.47
BMI before pregnancy [kg/m^2^]	23.7 (20.7–28.3)	23.2 (21.1–26.2)	24.6 (21.4–31.0)	0.57	0.29	0.22
BMI at birth [kg/m^2^]	28.3 (25.0–32.6)	27.6 (25.0–30.8)	28.2 (25.4–35.6)	0.47	0.34	0.20
BMI increase during pregnancy [kg/m^2^]	3.8 (2.7–5.3)	3.8 (2.8–5.0)	3.4 (2.5–4.9)	0.77	0.40	0.40
Newborn body mass [g]	3290 (2900–3600)	3340 (2970–3680)	3408 (3165–3683)	0.68	0.34	0.60
APGAR [0–10]	10.0 (10.0–10.0)	10.0 (10.0–10.0)	10.0 (10.0–10.0)	0.78	0.082	0.10

BMI—body mass index. IQR—Interquartile Range. **^&^** Mann–Whitney U test.

**Table 5:** Clinical parameters of women with GDM stratified according to *UBE2E2* rs6780569 genotype.

Parameters	*UBE2E2* rs6780569 Genotype					
	GG *n* = 176	GA *n* = 25	AA *n* = 3	GG vs. GA	GG vs. AA	GA vs. AA
	Median (IQR)	Median (IQR)	Median	*p* ^&^		
Fasting glucose [mg/dl]	99.0 (94.0–105.0)	97.0 (93.0–103.0)	94.0	0.55	0.91	0.91
Daily insulin requirement [unit]	0.0 (0.0–4.0)	0.0 (0.0–0.0)	0.0	0.22	0.98	0.62
Body mass before pregnancy [kg]	65.0 (56.5–76.0)	67.0 (59.0–70.0)	62.0	0.65	0.44	0.28
Body mass at birth [kg]	76.0 (67.0–89.0)	76.0 (67.0–89.0)	70.0	0.68	0.21	0.13
Body mass increase during pregnancy [kg]	10.5 (7.0–14.0)	12.0 (8.0–14.0)	7.0	0.44	0.17	0.12
BMI before pregnancy [kg/m^2^]	23.5 (20.9–27.9)	24.7 (21.0–28.4)	22.7	0.59	0.65	0.53
BMI at birth [kg/m^2^]	27.7 (25.0–31.8)	29.1 (25.4–33.6)	25.2	0.63	0.29	0.22
BMI increase during pregnancy [kg/m^2^]	3.7 (2.7–5.2)	4.2 (2.7–5.3)	3.1	0.50	0.21	0.17
Newborn body mass [g]	3333 (2910–3690)	3360 (3180–3530)	3100	0.72	0.45	0.19
APGAR [0–10]	10.0 (10.0–10.0)	10.0 (10.0–10.0)	10.0	0.17	0.50	0.73

BMI—body mass index. IQR—Interquartile Range. **^&^** Mann–Whitney U test.

**Table 6:** Clinical parameters of women with GDM stratified according to *WFS1* rs4689388 genotype.

Parameters	*WFS1* rs4689388 Genotype					
	AA *n* = 64	GA *n* = 96	GG *n* = 44	AA vs. GA	AA vs. GG	GA vs. GG
	Median (IQR)	Median (IQR)	Median (IQR)	*p* ^&^		
Fasting glucose [mg/dl]	97.0 (92.5–104.5)	99.0 (96.0–105.5)	99.0 (93.0–105.5)	0.20	0.49	0.74
Daily insulin requirement [unit]	0.0 (0.0–0.0)	0.0 (0.0–7.5)	0.0 (0.0–0.0)	0.36	0.88	0.42
Body mass before pregnancy [kg]	65.5 (57.0–72.0)	64.5 (56.0–77.5)	65.5 (58.0–76.0)	0.70	0.67	0.92
Body mass at birth [kg]	78.5 (68.0–87.5)	75.5 (67.0–89.5)	75.5 (68.0–88.5)	0.73	0.98	0.75
Body mass increase during pregnancy [kg]	11.0 (8.0–14.0)	10.0 (7.0–13.0)	10.0 (7.0–14.5)	0.14	0.42	0.78
BMI before pregnancy [kg/m^2^]	23.8 (21.3–26.8)	23.1 (20.6–28.4)	24.7 (22.1–28.3)	0.76	0.31	0.26
BMI at birth [kg/m^2^]	27.8 (25.4–32.1)	26.8 (24.7–32.7)	29.3 (25.7–31.0)	0.28	0.61	0.17
BMI increase during pregnancy [kg/m^2^]	4.1 (3.0–5.4)	3.6 (2.6–4.7)	3.7 (2.5–5.3)	0.090	0.53	0.51
Newborn body mass [g]	3225 (2768–3665)	3375 (3058–3665)	3368 (2960–3625)	0.20	0.36	0.86
APGAR [0–10]	10.0 (10.0–10.0)	10.0 (10.0–10.0)	10.0 (10.0–10.0)	0.45	0.17	0.39

BMI—body mass index. IQR—Interquartile Range. ^&^ Mann–Whitney U test.

## Data Availability

Not applicable.

## Abbreviations

95% CI	95% confidence interval
BMI	Body mass index
GDM	Gestational diabetes mellitus
HWE	Hardy–Weinberg equilibrium
IADPSG	International Association of Diabetes and Pregnancy Study Groups
IQR	Interquartile range
OGTT	Oral glucose tolerance test
OR	Odds ratio
PPARG	Peroxisome proliferator-activated receptors-γ
T2DM	Type 2 diabetes
TMEM163	Transmembrane Protein 163
*UBE2E2*	Ubiquitin Conjugating Enzyme E2 E2
*WFS1*	Wolframin ER Transmembrane Glycoprotein
